# A community effort towards a knowledge-base and mathematical model of the human pathogen *Salmonella *Typhimurium LT2

**DOI:** 10.1186/1752-0509-5-8

**Published:** 2011-01-18

**Authors:** Ines Thiele, Daniel R Hyduke, Benjamin Steeb, Guy Fankam, Douglas K Allen, Susanna Bazzani, Pep Charusanti, Feng-Chi Chen, Ronan MT Fleming, Chao A Hsiung, Sigrid CJ De Keersmaecker, Yu-Chieh Liao, Kathleen Marchal, Monica L Mo, Emre Özdemir, Anu Raghunathan, Jennifer L Reed, Sook-Il Shin, Sara Sigurbjörnsdóttir, Jonas Steinmann, Suresh Sudarsan, Neil Swainston, Inge M Thijs, Karsten Zengler, Bernhard O Palsson, Joshua N Adkins, Dirk Bumann

**Affiliations:** 1 Center for Systems Biology, University of Iceland, Reykjavik, Iceland; 2 Faculty of Industrial Engineering, Mechanical Engineering & Computer Science University of Iceland, Reykjavik, Iceland; 3 Department of Bioengineering, University of California, San Diego, La Jolla, CA, USA; 4 Infection Biology, Biozentrum, University of Basel, Basel, Switzerland; 5 USDA-ARS, Plant Genetics Research Unit, Donald Danforth Plant Science Center, St Louis, MO, USA; 6 Technical University Braunschweig, Institute for Bioinformatics & Biochemistry, Braunschweig, Germany; 7 Division of Biostatistics and Bioinformatics, Institute of Population Health Sciences, National Health Research Institutes, Zhunan, Taiwan; 8 Science Institute, University of Iceland, Reykjavik, Iceland; 9 Centre of Microbial and Plant Genetics, Department of Microbial & Molecular Systems, Katholieke Universiteit Leuven, Leuven, Belgium; 10 Laboratory of Computational Systems Biotechnology, Ecole Polytechnique Fédérale de Lausanne, Swiss Institute of Bioinformatics, Lausanne, Switzerland; 11 Department of Infectious Diseases, Mount Sinai School of Medicine, New York City, NY, USA; 12 Department of Chemical & Biological Engineering, University of Wisconsin-Madison, Madison, WI, USA; 13 Faculty of Life & Environmental Sciences, University of Iceland, Reykjavik, Iceland; 14 Department of Biochemical and Chemical Engineering, Technische Universität Dortmund, Dortmund, Germany; 15 School of Computer Science, The University of Manchester, Manchester, UK; 16 The Manchester Centre for Integrative Systems Biology, Manchester Interdisciplinary Biocentre, The University of Manchester, Manchester, UK; 17 Biological Sciences Division, Pacific Northwest National Laboratory, Richland, WA, USA

## Abstract

**Background:**

Metabolic reconstructions (MRs) are common denominators in systems biology and represent biochemical, genetic, and genomic (BiGG) knowledge-bases for target organisms by capturing currently available information in a consistent, structured manner. *Salmonella enterica *subspecies I serovar Typhimurium is a human pathogen, causes various diseases and its increasing antibiotic resistance poses a public health problem.

**Results:**

Here, we describe a community-driven effort, in which more than 20 experts in *S*. Typhimurium biology and systems biology collaborated to reconcile and expand the *S*. Typhimurium BiGG knowledge-base. The consensus MR was obtained starting from two independently developed MRs for *S*. Typhimurium. Key results of this reconstruction jamboree include i) development and implementation of a community-based workflow for MR annotation and reconciliation; ii) incorporation of thermodynamic information; and iii) use of the consensus MR to identify potential multi-target drug therapy approaches.

**Conclusion:**

Taken together, with the growing number of parallel MRs a structured, community-driven approach will be necessary to maximize quality while increasing adoption of MRs in experimental design and interpretation.

## Background

The evolution of antibiotic resistance by a variety of human pathogens is a looming public health threat [1,2]. *Salmonella *is a major human pathogen and a model organism for bacterial pathogenesis research [3]. *S. enterica *subspecies I serovar Typhimurium (*S*. Typhimurium) is the principle subspecies employed in molecular biology and its variants are causative agents in gastroenteritis in humans. The publication of the annotated genome for *S*. Typhimurium LT2 provided a foundation for numerous applications, such as drug discovery [4]. Previous efforts to systematically identify candidate drug targets within metabolism did not result in a plethora of new candidates, due to the robustness and redundancy of *S*. Typhimurium's metabolic network [5]. Since new single protein targets are missing, we need to target multiple proteins conjointly. Unfortunately, antibiotic regimens, which require multiple targets to be hit simultaneously, have an increased probability of the pathogen evolving resistance relative to a single target therapy. However, the continuous clinical success of the combination of beta-lactams and beta-lactamase inhibitors actually demonstrates that inhibitor combinations can be successful even if each individual inhibitor is non-effective on its own. The robustness inherent to *S*. Typhimurium's metabolic network imposes combinatorial challenges for *in vitro *and *in vivo *approaches to identify synthetic lethal genes sets (*i.e.*, experimental enumeration of all synthetic lethal pairs in *S*. Typhimurium would require the creation of ~500,000 double gene deletion strains, see below). Employing a systems biology network perspective could facilitate their identification.

GEnome scale Network REconstructions (GENRE) [6] represent biochemical, genetic, and genomic (BiGG) knowledge-bases [7] for target organisms; and have been developed for expression [8,9], metabolic [6,10], regulatory [11], and signaling [12,13] networks. Metabolic reconstructions (MRs) are the most developed out of the four GENRES. The metabolic network reconstruction process is well established [14] and has been used for various biotechnological and biomedical applications [15,16]. Given the rapidly growing interest in MRs and modeling, parallel reconstruction efforts for the same target organism have arisen and resulted in alternative MRs for a number of organisms [17-23]. These parallel MRs may vary in content and format due to differences in reconstruction approaches, literature interpretation, and domain expertise of the reconstructing group. Subsequent network comparison and discoveries are hampered by these differences. Consequently, the need for a community approach to divide the substantial effort required in reconciling and expanding these MRs has been formulated [17].

## Results and Discussion

### Salmonella, a reconstruction jamboree for an infectious disease agent

In June 2008, it became apparent that two MRs were being assembled by two different research groups [20] (Bumann, unpublished data). Subsequently, a *Salmonella *reconstruction jamboree was held at the University of Iceland, Reykjavik, from September 5th to 6th, 2008. The jamboree team consisted of over 20 experts in microbiology, proteomics, *Salmonella *physiology, and computational modeling. Based on the experience with the yeast reconstruction jamboree [17], a methodology was devised to increase the efficiency of community-based network reconstruction [24] and applied to the *Salmonella *reconstruction jamboree.

The goal of a network reconstruction jamboree is to provide a 2-D genome annotation that is of higher quality than it may be achieved by bioinformatic analyses alone [24,25]. The objective of this jamboree was to re-evaluate, reconcile, and expand the currently available MRs for *S*. Typhimurium with a focus on virulence. Furthermore, we aimed to include standard identifiers for reconstruction metabolites, reactions, and genes to facilitate subsequent mapping of 'omics' data. The starting MRs were AJRecon (a variant is published in [20]) and BRecon (D. Bumann, unpublished data), which were derived from published *E. coli *MRs, iJR904 [26] and iAF1260 [27], respectively, and their contents were modified to account for *Salmonella*-specific properties; i.e., transport and enzymatic reactions not present in *Salmonella *were removed and the proteins associated with the reactions were modified to contain proteins present in *S*. Typhimurium LT2.

#### Comparison of two metabolic reconstructions for *S*. Typhimurium

**Figure 1:**
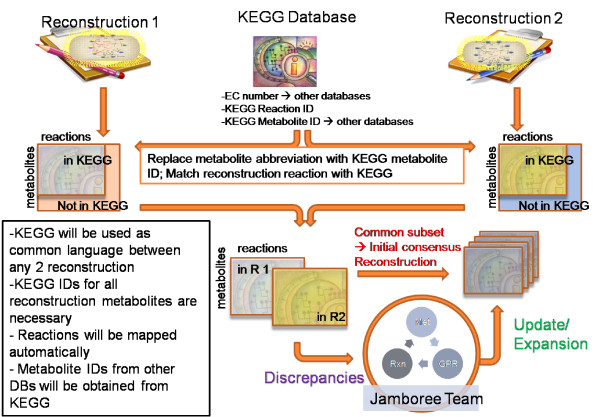
**Approach to reconcile two metabolic reconstructions (MR)**. This figure illustrates the automated comparison tool developed for the Salmonella reconstruction jamboree. Both MRs are translated into a common language (based on KEGG [44]). Metabolites and reactions that could not be mapped onto KEGG were subject to manual evaluation by the jamboree team. The overlapping part of the MRs was directly moved into the consensus MR while reactions and metabolites unique to a MR were evaluated manually. This approach can be readily applied to comparison of any two MRs.

We developed an automatic approach to initiate the reconciliation of the two MRs by converting their metabolites and reactions into a common language (Figure 1). The MR contents were grouped into three categories: (1) identical, (2) similar, and (3) dissimilar. A similar reaction was one, in which there was a minor discrepancy, such as reaction reversibility, a missing reactant or product, or a difference in associated enzyme(s). Dissimilar reactions were those with distinct sets of reactants and products, and often represented metabolic reactions that were not included in one of the starting MRS. The identical content was transferred to the consensus MR without further evaluation. The similar and dissimilar content was evaluated at the jamboree. Genes and proteins associated with the reactions were also carefully compared and refined where necessary. At its end, the meeting yielded an approximately 80% reconciled consensus reconstruction. The remaining discrepancies were manually curated by the Bumann and Palsson groups following the jamboree meeting.

Initial comparison revealed that there were 760 reactions common to the starting MRs while 521 and 1684 reactions were unique to AJRecon and BRecon, respectively (Additional file 1 Table S1). Some of these differences could be explained by changes introduced to *the E. coli *MR when it was converted from its earlier version, iJR904 [26], to the most recent version, iAF1260 [27] (i.e., explicit definition of a periplasm compartment; more detailed fatty acid metabolism).

### Characteristics of the Salmonella Consensus Reconstruction

The resulting knowledge-base, STM_v1.0 (Table 1; Additional file 2; Additional file 1 Table S2), represents the final product of a community-effort to develop a detailed MR of *S*. Typhimurium. STM_v1.0 integrates the novel and common features of the starting MRs into a vetted, well-documented consensus knowledge-base, capturing currently available BiGG knowledge about *S*. Typhimurium. Key features of STM_v1.0 include i) accounting for the periplasm as a compartment between the extracellular space and cytoplasm; ii) *Salmonella*-specific virulence characteristics, such as iron chelation by salmochelin and serovar Typhimurium LT2 O-antigen production; iii) the possibility to employ the consensus MR as mathematical, predictive model; and iv) comprehensive support data for reactions and associated genes (Additional file 1 Table S2a). Some information was excluded from STM_v1.0, such as the 26 dipeptide and tripeptide transport/digestion reactions that are present in AJRecon, as they represent generic compounds. Accounting for all potential consumable oligopeptides would make computational analysis intractable or unnecessarily difficult. Appropriate oligopeptides may be manually added to STM_v1.0 to represent a specific growth environment. We also attempted to exclude reactions that were included to fit some growth data [28], but were contrary to other observations [20,29] as was the case for growth with D-aspartic acid [30] as the sole carbon source which requires an unknown transporter and an unknown aspartate racemase [31].

**Table 1:** Basic Statistics for the original and the consensus reconstructions.

	**AJRecon**[20]	BRecon	iMA945* [21]	Consensus (new data)
Genes	1,119	1,222	945	1,270

Network reactions	1,079	2,108	1,964	2,201

-Transport reactions	200	575	726	738

Biochemical reactions	879	1,533	1,238	1,463

Metabolites (unique)	754	1,084	1,035	1,119

Compartments	Cytosol, extracellular space	Cytosol, periplasm, extracellular space	Cytosol, periplasm, extracellular space	Cytosol, periplasm, extracellular space

* Not included in consensus reconstruction. See text for details.

Additionally, we evaluated the reaction directionality of consensus MR reactions by considering thermodynamic properties of participating metabolites. In the case that a thermodynamic prediction was inconsistent with experimental evidence, the experimental evidence was followed. Thermodynamic predictions are made using the knowledge that is available [45], and incorrect predictions highlight gaps in our knowledge of biology.

A bacterial MR often includes a biomass reaction that lists all known biomass precursors and their fractional contribution necessary to produce a new bacterial cell in a given environment. The individual biomass constituents of a *S*. Typhimurium cell have been measured [20], and adapted for the consensus reconstruction by accounting for the changes in naming and compartments introduced during reconciliation (Additional file 1 Table S3c).

#### Comparison with a third metabolic reconstruction of *S*. Typhimurium

After finishing the consensus reconstruction, a third metabolic reconstruction (iMA945) was published [21]. Similar to one of our starting MRs (BRecon), iMA945 was built by using homology, and other bioinformatics criteria [32], starting from the *E. coli *metabolic reconstruction (iAF1260). Gaps in iMA945 were detected and filled with GapFind and GapFill, respectively [33]; and iMA945's content was further augmented by the GrowMatch algorithm [34] to fit experimental measurements. These automated optimization methods are excellent tools for identifying gaps in network reconstructions and proposing candidate reactions to fill these gaps and fit the model to growth data, however, they often do not associate genes with the candidate reactions. The candidate reactions are typically taken from a universal reaction database (such as KEGG) that includes pathways from all domains of life, thus candidate reactions proposed by these methods should be taken as hypotheses and require additional validation from published literature or direct experimental evidence.

We performed a preliminary comparison between STM_v1.0 and iMA945. However, we did not reconcile iMA945 with the consensus reconstruction, as this will require detailed evaluation of the discrepancies in a subsequent jamboree meeting. Overall, 2,057 reactions were present in both the consensus reconstruction and iMA945, of which 1,706 reactions have identical gene-protein-reaction (GPR) associations (Additional file 1 Table S2d). A total of 26 reactions had identical reaction identifiers but different reactions (*e.g.*, different reactants, products, stoichiometry, or directionality: reversible, forward only, backward only) and GPR associations. There were a total of 629 distinct reaction ids between STM_v1.0 and iMA945: 446 were unique to STM_v1.0 and 183 to iMA945. Of the 183 reactions flagged as unique to iMA945, the majority represents reactions that were intentionally excluded from the consensus reaction (*e.g.*, 45 dipeptide exchange, transport, and peptidase reactions and >60 additional exchange, transport, and enzymatic reactions not supported by literature). Some of the distinct reactions, such as adenosylcobalamin phosphate synthase, were due to different metabolite and reaction identifiers. No bibliomic data were included in iMA945, so it was not possible to assess whether the reactions were inserted by the automated gap-filling methods or supported by additional evidence. The 446 reactions unique to STM_v1.0 include *Salmonella-*specific chelators, O-antigens, and lipid modifications that were not present in the starting network derived from the *E. coli *MR (iAF1260). Overall, the core metabolic network is similar between STM_v1.0 and iMA945, which is expected as the draft scaffolds for both MRs were derived from *E. coli *MRs and *S*. Typhimurium has a notable metabolic homology with *E. coli*; however, STM_v1.0 includes over 300 more genes than iMA945 and includes a variety of *Salmonella*-specific reactions that are essential for virulence and could serve as coupling points for constructing a host-pathogen model.

### Metabolic Network Reconstruction Assessment

To assess the utility of a mathematical approximation of reality, it is essential to determine the consistency of the model's predictions with real-world benchmarks. In the case of MRs, comparing experimental growth data with predicted biomass production is a commonly employed metric in benchmarking metabolic models [14]. Although biomass production is a commonly employed metric, the results should always be taken with a grain of salt; for instance, it is possible to improve the fitting of a model's predictions to growth data by including enzymatic reactions for which no evidence exists or which are contrary to published experimental observations. The reconstruction committee chose not to include invalidated enzymatic reactions that improved the fit between growth predictions and experimental observations; the failings of the model's predictions highlight areas where knowledge is lacking and experimental undertakings could identify new knowledge.

For *S*. Typhimurium, there is a wealth of experimental growth data [29]. Overall, we found good agreement between the qualitative growth phenotype predictions and the experimental data (Table 2 Additional file 1 Table S4); with the notable exception of sulfur metabolism where the prediction accuracy was about 40%. As we are becoming increasingly aware of the importance of sulfur-related metabolism in host-pathogen interactions [35-38], the deficiencies in our knowledge highlighted by this analysis represent viable targets for experimental enquiry. For the carbon and nitrogen sources accessible by AJRecon our results were comparable, however STM_v1.0 has the ability to metabolize 20 carbon sources and 15 nitrogen sources not accessible to AJRecon. The additional metabolic capabilities of STM_v1.0 are due, in part, to the presence of ~200 additional gene products in STM_v1.0.

**Table 2:** Growth benchmark results

		Experiment	
Source (accuracy)	Prediction	Growth	No Growth
Carbon	Growth	79	9
(118/133)	No Growth	6	39

Nitrogen	Growth	28	5
(57/64)	No Growth	2	29

Phosphate	Growth	24	0
(24/25)	No Growth	1	0

Sulfur	Growth	6	0
(8/14)	No Growth	6	2

### Gene Essentiality Simulations

To combat the rise in antibiotic-resistant pathogens, it is crucial to identify new drug targets. Genes or sets of genes that are essential for growth are potential drug targets. To identify novel drug targets in STM_v1.0, we performed single and double gene deletion studies. We identified 201 essential genes in M9/glc, 144 of which were also essential in LB (Additional file 1 Table S5a). The synthetic lethal gene pair simulations were performed using only genes that were found to be non-essential in the condition of interest (Additional file 1 Table S6). In M9/glc, there were 87 synthetic lethal gene-pairs comprised of 102 unique genes. For *E. coli*, Suthers *et al. *[39] predicted 86 synthetic lethal gene-pairs, however, there were only 83 unique genes involved. In LB, there were 56 synthetic lethal gene-pairs comprised of 76 unique genes. Interestingly, 10 of LB synthetic lethal genes were also essential in M9/glc and were members of 12 of the LB synthetic lethal gene-pairs. The very small fraction of essential synthetic lethal gene pairs (< 100 synthetic lethalities out of >500,000 possibilities - assuming approx. 1000 non-essential metabolic genes) emphasizes the robustness of *S*. Typhimurium's metabolic network, which has previously been noted [5].

### Candidate drug targets

Our observed, very small number of synthetic lethal pairs in STM_v1.0 indicates that antimicrobial regimens may need to target more than two elements to be effective. Unfortunately, it will take less time for a pathogen to evolve a solution to a conjoint two-target antimicrobial strategy compared to a single-target strategy. To reduce the probability of a pathogen evolving resistance to a conjoint two-target strategy, it may be plausible to employ a combination of two-target strategies. Although a combination approach may be suitable for dealing with antibiotic resistance, there are potential shortcomings associated with clearance and toxicity because all the components of a regimen must reach a target at a specific time with the requisite concentrations. Despite these difficulties, multi-component, multi-target drugs are becoming standard therapeutics for complex diseases, including cancer, diabetes, and infectious diseases [40]. Experimental identification and characterization of therapeutic strategies that require multiple targets for effectiveness is a resource intensive undertaking (*e.g.*, creating over 500,000 double mutant strains). An *in silico *approach using an MR, such as STM_v1.0, could be implemented to prioritize the experiments by indicating which multi-target therapies would adversely affect the pathogen's metabolic capabilities.

**Figure 2:**
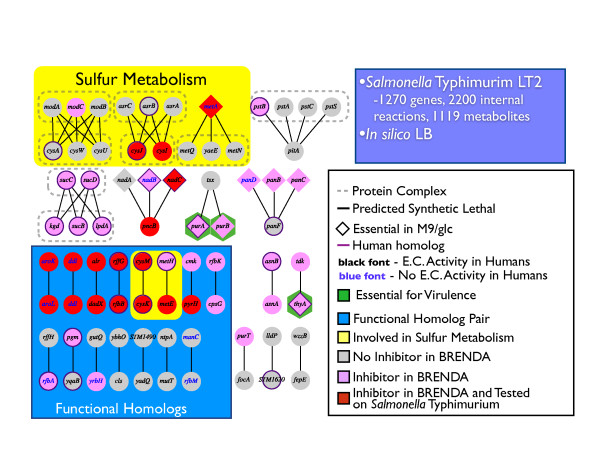
**Candidate drug targets**. The figure contains all predicted synthetic lethal interactions for STM_v1.0 in LB medium. A line connecting two genes represents a synthetic lethal pair. A group of genes surrounded by a dashed box represents a protein complex requiring all enclosed elements to function. Yellow background means associated with sulfur metabolism. Blue background indicates that the synthetic lethal pairs are functional homolog's. Red gene means that there is a chemical known to inhibit the gene-product in STM_v1.0.

As mentioned above, the synthetic gene deletion analysis yielded 56 synthetic lethal gene pairs disrupting growth of *S*. Typhimurium *in silico*. We grouped these gene pairs based on different criteria to assess their potential value as multi-drug targets (Figure 2). It is notable that five gene pairs are between protein complexes while a further three gene pairs are between genes involved in the same pathway - this indicates the presence of a layer of 'redundancy' for the enzyme or pathway that confers protection against a single-target therapy. Moreover, three of the genes involved in gene pairs are known to be essential for virulence, but not for growth, and have known inhibitors based on BRENDA [41]. This structured overview of *in silico *synthetic lethal gene pairs identified numerous candidate drug targets many of which have known inhibitors. In subsequent studies, these model-generated hypotheses need to be tested and validated.

Additional gene products shown to play a central role in virulence yet are not essential for growth in laboratory conditions or do not have an unequivocal functional annotation represent additional therapeutic targets. These gene products could serve as potential points for manipulating host metabolism [38], could be essential for metabolism in the host environment (*e.g., Salmonella*-containing vacuoles are nutrient poor)[42], and will represent an energy and materials demand when creating integrated metabolic and expression reconstructions [8,9]. Recent examples of relevant gene products that have not been annotated but are crucial for virulence include gene products STM3117-STM3120 [43]; as the metabolic functions of unannotated genes are elucidated, they will need to be incorporated into future revisions of the MR.

## Conclusions

Taken together, the community-developed consensus MR is a curated reconstruction with the combined properties of the starting MRs and new information that was added during and after the reconstruction jamboree. The expanded metabolic versatility with a focus on virulence, updated annotation, including corrections, and curation of hundreds of additional reactions, genes, and metabolites by a community of experts present in STM_v1.0 highlights the value of a community-based approach. Another MR for *S*. Typhimurium was published after the jamboree [21], which was also based on an *E. coli *MR[27]. The reconciliation with this third MR will need to be done in subsequent jamboree meetings, which will also lead to a further expansion of knowledge and data included in the consensus knowledge-base. The publication of the third MR for *S*. Typhimurium emphasizes the importance and the value of the effort presented in this report as well as the need for additional outreach when assembling jamboree committees.

## Methods

### Metabolic network reconstructions of Salmonella enterica serovar *Typhimurium LT2*

The starting reconstructions, AJRecon and BRecon, were built on scaffolds derived from published *E. coli *MRS. AJRecon is a pre-publication version of iRR1083 [20], and was based on iJR904 [26]. For its scaffold, BRecon (Bumann, unpublished) employed iAF1260 [27]- a direct descendent of iJR904. The two reconstructions, differ in content due to: (1) different components being targeted for manual curation (e.g., BRecon extended Fe chelation and AJRecon extended lipid production), and (2) differences in *E. coli *MRs that were used as comparative genomics scaffolds for initializing the *Salmonella *MRs (e.g., iAF1260 accounted for the periplasm whereas its ancestor did not).

### Method for community-based network reconstruction

There are three essential phases for community-based MR development: (1) preparation, (2) jamboree, and (3) reconstruction finalization [24]. The preparation and finalization phases are carried out by a small contingent of researchers, whereas, the collective knowledge of the community is harnessed during the jamboree. In the preparation phase, the two MRs were compared as described below in terms of metabolites, reactions, and gene-protein-reaction associations (GPRs). Overlapping content between both original MRs was directly moved into the consensus MR (Additional file 1 Table S1). Discrepancies in the listed three areas were presented to the jamboree team, which was split into three groups: metabolite curation, reaction curation group, and GPR curation group. The metabolites group curated the list of all metabolites present in either original MR for i) protonation state of metabolites at physiological pH, ii) missing metabolite identifiers: KEGGID, PubChemID, ChEBI ID, and iii) comparison of neutral formulae in reconstruction and metabolite databases. The reaction group was responsible for identifying evidence for orphan reactions in either original MR with and without a KEGG reaction ID. Reactions without a KEGG ID had to be extensively audited as there were no database evidences for the correctness of the reaction mechanisms. The GPR group had to resolve the discrepancies in GPR assignments using genome databases and literature. Each team evaluates their problem set based on evidence within the consensus MR and available resources (literature, databases, and annotations). Items that are not adequately addressed during the jamboree are subject to extensive manual curation during the MR finalization phase. The finalization phase includes: (1) manual curation, (2) benchmarking the consensus MR against experimentally-derived phenotypic data, and (3) MR dissemination. The consensus MR is expected to be maintained, updated and expanded in subsequent reconstruction jamborees.

### Metabolic Reconstruction Reconciliation

Reconciling multiple MRs requires that the MRs' contents employ a common nomenclature so that the contents may be compared. For this work, we employed the KEGG database [44] as the source of common identifiers (Figure 1); although all of the reactions and metabolites in KEGG may not be accurate or complete, KEGG has the benefit of being an extensive, freely accessible resource used by the broader biological community. The complete consensus reconstruction can be found in Additional file 1 Table S6 and in Additional file 2 as an SBML file.

### Thermodynamic directionality

Thermodynamic directionality for each reaction was calculated as described in [45]. Briefly, assuming a temperature of at 310.15 K, intracellular pH of 7.7, extracellular/periplasmic pH of 7.0, and a concentration range of 0.01-20 mM, we calculated upper and lower bounds on transformed reaction Gibbs energy, and assigned reaction directionality accordingly. Transport reactions were not subject to thermodynamic consistency analysis as there is still uncertainty associated with the directionality prediction of transmembrane transport.

### Conversion of reconstruction into a mathematical model

The conversion of a reconstruction into a mathematical model has been described in detail elsewhere [14]. The unit of reaction fluxes was defined as mmol/g_DW_/hr.

### Phenotypic assessment

Flux balance analysis [46] was employed to assess the STM_v1.0 model's ability to correctly predict biomass production in a variety of limiting conditions. The accuracy of the model was assessed by comparing the predictions to benchmarks drawn from experimental data [20,29]. In this assessment, there are four possible observations: (1) STM_v1.0 model correctly predicts growth (G/G), (2) STM_v1.0 model incorrectly predicts growth (G/NG), (3) STM_v1.0 model correctly predicts no growth (NG/NG), and (4) STM_v1.0 model incorrectly predicts no growth (NG/G). For a prediction to be counted as a true positive (G/G) or true negative (NG/NG), the prediction needed to match one or more experimental observations. The predictions were first compared with the Biolog phenotype microarray (PM) data http://www.biolog.com. False positive predictions (G/NG) and false negative predictions (NG/G) were then compared with the data from Gutnick *et al. *[29] and references cited in Ragunathan *et al. *[20]. For limiting conditions not represented in the PM, predictions were only compared with data from Gutnick *et al. *[29] or cited in Ragunathan *et al. *[20].

### Gene essentiality analysis

The gene deletion studies were performed by converting STM_v1.0 into a stoichiometric model and performing flux balance analysis [46]. For each gene, or gene pair, the associated reaction(s) were disabled (v_min, i _= v_max, i _= 0 mmol.gDW^-1^.hr^-1^) and the ability of the model to produce biomass was assessed, i.e., the biomass reaction was chosen as the objective function and maximized.

All simulations were performed using the COBRA Toolbox v2.0 [47] using Matlab (Mathworks, Inc) as the programming environment, and Tomlab (TomOpt, Inc) as the linear programming solver.

## Authors' contributions

IT, DRH, BOP, JNA, and DB conceived the study. BS and DB compiled the BRecon. IT and DRH compiled the consensus MR. IT, DRH, BOP, and DB wrote the manuscript. GF and IT designed and performed initial MR comparisons. RMTF and DRH performed thermodynamic directionality analysis. DHR and IT carried out the computational analysis of the consensus MR. IT, BOP, DB, BS, DKA, SB, PC, FCC, RMTF, CAH, SCJK, YCL, KM, MLM, EÖ, AR, JLR, SIS, SS, JS, SS, NS, IMT, KZ, BOP, JNA, DB actively participated during and/or after the metabolic reconstruction jamboree to generate content for the consensus MR. All authors read and approved the final manuscript.

## Supplementary Material

Additional file 1 **Consensus MR**. This xlsx file contains the consensus reconstruction and simulation setup/results. - Table S1. Statistics for automated reconciliation of starting reconstructions. - Table S2. Consensus Reconstruction in SBML format. - Table S3a. M9/glc. - Table S3b. LB. - Table S3c. Biomass. - Table S4. Growth benchmark errors. - Table S5a. All Lethal deletion predictions. - Table S5b. Single Deletion/Virulence. - Table S6a. LB Synthetic Lethal. - Table S6b. M9 Synthetic Lethal Click here for file

Additional file 2 **Consensus MR in SBML format**. Consensus MR as a computational model in SBML format. Click here for file
